# Robust Visual Tracking Using Structural Patch Response Map Fusion Based on Complementary Correlation Filter and Color Histogram

**DOI:** 10.3390/s19194178

**Published:** 2019-09-26

**Authors:** Zhaohui Hao, Guixi Liu, Jiayu Gao, Haoyang Zhang

**Affiliations:** 1 School of Mechano-Electronic Engineering, Xidian University, Xi’an 710071, Shaanxi, China; haozhaohui@stu.xidian.edu.cn (Z.H.); zhanghy@stu.xidian.edu.cn (H.Z.); 2 Shaanxi Key Laboratory of Integrated and Intelligent Navigation, Xi’an 710068, Shaanxi, China; gaojiayu_cool@126.com; 3 Xi’an Research Institute of Navigation Technology, Xi’an 710068, Shaanxi, China

**Keywords:** visual tracking, correlation filter, color histogram, adaptive hedge algorithm

## Abstract

A part-based strategy has been applied to visual tracking with demonstrated success in recent years. Different from most existing part-based methods that only employ one type of tracking representation model, in this paper, we propose an effective complementary tracker based on structural patch response fusion under correlation filter and color histogram models. The proposed method includes two component trackers with complementary merits to adaptively handle illumination variation and deformation. To identify and take full advantage of reliable patches, we present an adaptive hedge algorithm to hedge the responses of patches into a more credible one in each component tracker. In addition, we design different loss metrics of tracked patches in two components to be applied in the proposed hedge algorithm. Finally, we selectively combine the two component trackers at the response maps level with different merging factors according to the confidence of each component tracker. Extensive experimental evaluations on OTB2013, OTB2015, and VOT2016 datasets show outstanding performance of the proposed algorithm contrasted with some state-of-the-art trackers.

## 1. Introduction

Visual object tracking is a fundamental research task and plays a crucial role in numerous computer vision applications including motion analysis, surveillance, segmentation, and autonomous driving and so forth [1]. Basically, the purpose of visual tracking is to estimate the motion trajectory of the target over successive video frames, only initializing its state at the first frame. Numerous robust tracking algorithms [2,3,4] have emerged and taken exciting progress gains in recent years. However, it is still a very challenging task to design a robust tracking algorithm due to significant target appearance variation caused by factors such as fast motion, shape deformation, partial occlusion, illumination change, background clutter, and so on. To overcome these issues, a more discriminative appearance representation which is a key part of successful tracking is needed.

Recently, tracking approaches based on discriminative correlation filters (DCFs) [5,6,7,8,9] have attracted considerable attentions and obtained excellent performances on several tracking benchmark datasets [10,11,12]. Benefited from the circular assumption of training samples, the DCFs-based algorithms can be learned and detected very efficiently in the Fourier domain by element-wise multiplication and, hence, is of significance for real-time tracking application. However, as traditional DCFs that use histogram of oriented gradients (HOG) features [13] strongly depend on the spatial layout of the tracked object, it is hard for them to handle deformation and rotation well.

**Figure 1:**
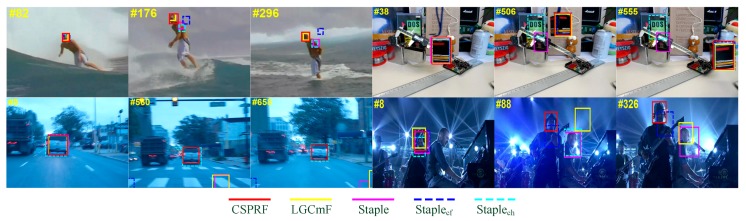
Tracking results of the Staple, its component trackers including Staple_cf_ and Staple_ch_, the LGCmF and our CSPRF tracker on four sequences. The Staple tracks failure on all four sequences and the LGCmF can track two of the four sequences, which illustrates the LGCmF indeed has some improvement. Our CSPRF can perform well on four sequences. From left to right and from top to bottom are Surfer, Box, BlurCar1, and Shaking sequences.

To tackle the above shortcoming, an effective tracker termed as Staple [14] has been proposed to compensate for the deficiencies of both color histograms and DCFs via linearly combining their response maps, which successfully deals with deformation and illumination variation simultaneously. However, there emerge two principal lacks of the Staple tracker. Firstly, the Staple tracker only employs holistic appearance representations of color histogram and DCFs, ignoring the underlying spatial local structural information, thereby its component trackers Staple_ch_ (only applying color histogram-based tracker) and Staple_cf_ (only applying DCFs-based tracker), are likely to perform poorly alone in some challenging scenarios such as partial occlusion and drastic deformation. This always leads to failure due to the merged inaccurate response maps. Secondly, Staple tracker resorts to a fixed merging percentage factor (i.e., 0.3) for overall performance on datasets, which may cause tracking failure because of considering too much unreliable component trackers in some complex scenes. Figure 1 illustrates the tracking results on four sequences to explain the above findings of the Staple tracker. Due to the failures of both Staple_cf_ and Staple_ch_ at frame 176 and frame 88 in Surfer and Shaking sequences respectively, Staple which is the result of merging these two components fails at these instants as well. Staple_cf_ fails at the 560th frame of the BlurCar1 sequence and Staple_ch_ fails at the 506th frame of the Box sequence. These tracking failures also lead to the failure of Staple tracker since it has no emphasis on reliable component tracker. The LGCmF tracker [15], which is an improved method based on the Staple, performs well on both Surfer and Box sequences, while fails on the BlurCar1 and Shaking sequences.

To alleviate the aforementioned deficiencies, in this work, we follow the research line of merging response maps between color histograms and DCFs [14] and embed a spatial local patch-structured framework to it for visual tracking. We first construct two part-based component trackers: correlation filter-based structural patch tracker (CFSP) and color histogram-based structural patch tracker (CHSP). In each of them, an adaptive hedge algorithm is introduced to determine weights of structural patches. The standard hedge algorithm [16] is an online decision theoretical method for multi-expert, which uses the difference between the loss of an expert and the weighted average loss of all experts to define the regret of this expert. This algorithm uses the cumulative regret corresponding to each expert to generate its weight in each frame. In this work, we treat each tracked patch as an expert and design a reliable loss metric for each expert by analyzing the similarity or the discrimination of these patches and the difference among displacement of each patch with the target. Then based on the tracking reliabilities of CFSP and CHSP, we selectively combine the response maps of them to formulate the final complementary structural patches response fusion tracker (CSPRF). Inspired by [15,17], we train and update a SVM detector for determining the confidences of the component trackers CFSP and CHSP, and implement a re-detect procedure when both of the component trackers are unreliable. From Figure 1, it can be seen that our proposed CSPRF tracker performs favorably when the Staple and LGCmF lose the target.

The main contributions of this work can be summarized as: (1) In contrast to existing Staple tracker which only uses holistic appearance representations of color histograms and DCFs, we use the local structural appearance information and propose an novel structural patch response map fusion tracking algorithm using complementary correlation filter and color histogram. (2) We develop an adaptive hedge algorithm for part-based tracking framework by adaptively considering the proportion of instantaneous and cumulative regrets of each expert over time. (3) We design two reliable loss measurement methods in correlation filter and color histogram models to provide credible inputs for the adaptive hedge algorithm, by which the correlation filter-based structural patch tracker (CFSP) and the color histogram-base structural patch tracker (CHSP) are proposed. (4) We execute the extensive experiments on three tracking benchmark datasets OTB2013 [10], OTB2015 [11], and VOT2016 [12] to demonstrate the efficiency and robustness of our proposed CSPRF tracker in comparison with the state-of-the-art trackers.

## 2. Related Work

### 2.1. Correlation Filter-Based Tracking

Since discriminative correlation filter-based tracking method was initially proposed by Bolme et al. [5], now it has been widely applied to the visual object tracking community and has been demonstrated very impressive performance on benchmark datasets. The work in [5] optimizes a minimum output sum of squared error filter (MOSSE) that uses simple grayscale features to represent the target appearance. According to the circular matrix structure and kernel trick, Henriques et al. [18] propose the circular structure with kernels tracking algorithm (CSK), and soon after extend this work to handle multi-channel features such as histograms of oriented gradients (HOG) [13], namely kernelized correlation filters (KCF) [6]. Danelljan et al. [19] introduce color names feature [20] to correlation filter for improving tracking performance. To resolve scale changing problem during tracking process, Danelljan et al. propose the DSST tracker [21] with a separate multi-scale correlation filter. To mitigate the boundary effects, Danelljan et al. propose the SRDCF tracker [7] by using a spatially regularized weight to penalize filter coefficients far away from the target center. Li et al. present spatial-temporal regularized correlation filters (STRCF) [9] by introducing temporal regularization to SRDCF. Additionally, Yang et al. present parallel correlation filters (PCF) [22] for visual tracking by constructing two parallel correlation filters. Zhang et al. propose a novel motion-aware correlation filters (MACF) tracking algorithm [23], which integrates instantaneous motion estimation Kalman filters into the correlation filters.

### 2.2. Color Histogram-Based Tracking

Color histograms [24,25,26] are a common method to model the object appearance representation among earlier tracking approaches. Compared to others features such as HOG or pixels, color histogram is robust to shape deformation and rotation, hence it is meaningful to track non-rigid objects. The early mean shift tracker [24] minimizes the Bhattacharyya distance of color histograms between the target object and the reference regions iteratively. Abdelai et al. [25] present an efficient accept–reject color histogram-based scheme embedding integral image into a Bhattacharyya kernel to find most similar area with target. Duffner et al. [26] construct a probabilistic segmentation using back-projection maps between foreground and background, where the target tracking process is accomplish by applying a generalized Hough transform with pixel-based descriptors. The distractor-aware tracker (DAT) [27] proposed by Possegger et al. formulates an efficient discriminative color histograms model to identify potentially distracters and significantly reduce the risk of drifting.

In recent years, there appear complementary learners [14,28] combining color histogram and correlation filter to represent the target, which are able to compensate each other in visual tracking. In [14], Bertinetto et al. linearly incorporate the output response maps of color histograms and correlation filters to achieve high tracking performance and speed. Fan et al. [29] present a dual color clustering and spatio-temporal regularized correlation regressions-based complementary tracker, where a color clustering-based histogram and a spatio-temporal regularized correlation filters are formulated as complementary learners to improve the tracking performance of [14]. Lukezic et al. [28] construct a spatial reliability map to adjust the filter support to the part of the target object suitable for tracking by exploiting color histograms. Zhang et al. [15] propose a collaborative local-global layer visual tracking method (LGCmF), in which a block tracker (SLC) utilizing structural local color histograms feature and a global correlation filter tracker based on HOG feature are merged in the response map level. Inspired by [15], the block strategy also is adopted in this work. In contrast to [15] that only applies part-based tracking strategy in color histogram model, we employ more complete blocking strategy in both component trackers and more efficient block weighting method for each patch based on adaptive hedge algorithm.

### 2.3. Part-Based Tracking

Part-based tracking algorithms focus on the local parts of the target and, hence, they are very robust to handle partial occlusion and severe deformation. Commonly, the visible parts can still provide reliable cues for tracking when the target is partially occluded. Nejhum et al. [30] match the intensity histograms of foreground blocks by dividing the foreground shape as several rectangular blocks to update the target shape and adjust layout of them. Zhang et al. [31] propose a part matching tracker (PMT) based on a locality-constrained low-rank sparse learning method to optimize partial permutation matrixes for image blocks among multiple frames. Yao et al. [32] present a latent structured learning method to model the unknown parts of target.

Several recent tracking methods have attempted to integrate the correlation filters into a part-based framework for improving the tracking performance [33,34]. Liu et al. [33] propose a part-based tracker with multiple adaptive correlation filters, where the Bayesian inference framework and a structural constraint mask are adopted to be robust to partial occlusion and deformation. Li et al. [34] identify the reliability of patches according to the motion trajectory and trackability of each patch. Sun et al. [35] present a shape-preserved kernelized correlation filter within a level set framework for deformable tracking of individual patches. Wang et al. [36] formulate an occlusion-aware part-based tracker that can convert between the global model and local model adaptively to avoid polluting target templates by background information.

### 2.4. Sparse-Based Tracking and Deep Learning-Based Tracking

In addition to correlation filter tracking and color tracking, popular tracking algorithms in recent years include sparse tracking [37,38,39] and deep learning tracking [40,41,42,43] as well. In sparse tracking, Zhang et al. [37] propose a novel sparse tracking method by matching framework for robust tracking based on basis matching. Zhang et al. [38] propose a tracker using a semi-supervised appearance dictionary learning method. Zhang et al. [39] develop a biologically inspired appearance model for robust visual tracking. As for deep learning based tracking, the work of [40] learns multi-level correlation filters with hierarchical convolutional features to integrate the correlation responses proportionally. Subsequently, Qi et al. [41] exploit an adaptive hedge algorithm to make a weighted decision of all weak correlation filter trackers. Zhang et al. [42] integrate the point-to-set distance metric learning (DML) into visual tracking tasks and take full advantage of all the training samples when determining the best target candidate. Danelljan et al. [43] introduce a novel tracking architecture consisting of two components designed exclusively for target estimation and classification. This method achieves a considerable performance gain against the previous tracking approach.

## 3. Proposed Algorithm

### 3.1. Overview

Following the Staple [14], our work also relies on the strengths of both correlation filters and color histograms. However, the Staple employing the holistic appearance information is likely to drift or fail in the scenes of severe deformation or partial occlusion. A part-based tracking strategy can achieve favorable tracking results for above challenging scenes, since reliable cues for tracking can be provided by remaining visible parts or undeformed parts. Therefore, in this work we take into account the structural local information of both correlation filters and color histograms, which show promising tracking performance improvement over Staple.

**Figure 2:**
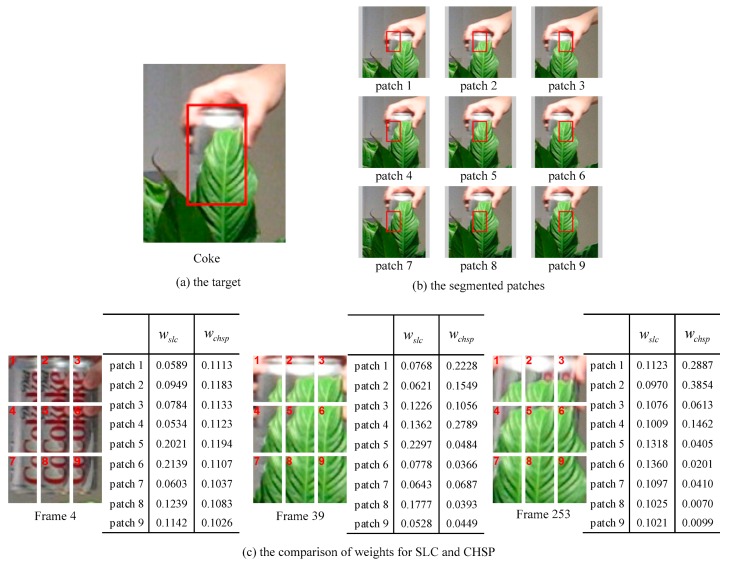
The segmented instance and the comparison of weights of patches in SLC and CHSP. (**a**) shows the tracking target with red bounding box in Coke sequence. (**b**) shows that the target is divided into nine overlapped patches using red bounding box. In (**c**), the tables list the patch’s weights of SLC and CHSP for the frames 4, 39, and 253. $w_{slc}$ and $w_{chsp}$ represent the weights of corresponding patches of SLC and CHSP, respectively.

Since the trackability of individual patches is distinct in different scenes, it should be highlighted for these patches with high trackability. The LGCmF [15] calculates the discrimination value to determine the trackability of individual patch in its component tracker SLC by the foreground-background discrimination analysis, which only considers the appearance information of the individual patch. To fully utilize both appearance discrimination and spatial motion information, we do not only consider discrimination value, but also allow motion consistency of individual patch with the target. And according to them, we formulate the loss metric of each patch tracker in CHSP, which is used as input for adaptive hedge algorithm. Figure 2c illustrates that our component tracker CHSP allocates more desirable weights to individual patch trackers than the SLC [15]. For instance the weights of our CHSP are more uniform than them of SLC when all patches are clearly visible at frame 4. And when the target is partially occluded at frames 39 and 253, the remaining visible patches 1, 2, 3, and 4 still can provide reliable tracking cues, which mean these patches are more likely to be tracked correctly, hence these patches are given higher weights in our CHSP.

In CFSP, we calculate the loss of individual patch tracker according to similarity and motion consistency of individual patch. The motion consistency refers to the difference among displacement of individual patch with the displacement of predicted target. And the similarity of each patch is measured by employing the intensity and the smooth constraint of response map between patch expert trackers.

**Figure 3:**
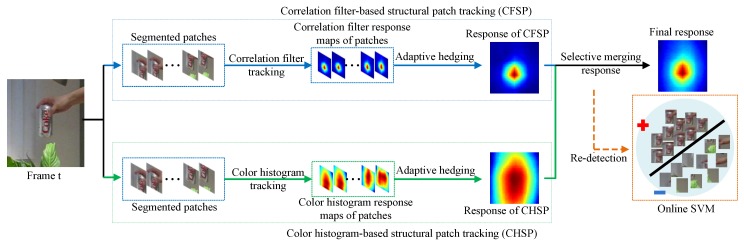
The flow chart of the proposed tracking algorithm. When a new frame t arrives, we first divide the target into several overlapped patches. The responses of correlation filter and color histogram of all these patches are computed. These correlation filter responses are combined together by the adaptive hedge algorithm to constitute the component tracker CFSP. With the same way, the component tracker CHSP is also constructed. Finally, responses of CFSP and CHSP are selectively fused to obtain final response and the new location of the target is estimated at its peak. When both of the combined responses are unreliable, an online SVM classifier is activated to re-detect the target.

We describe the main steps of the proposed approach in Figure 3. In this section, we first present the adaptive hedge algorithm, and then describe the two component trackers in detail. Based on these component trackers, we formulate the final complementary structural patches response fusion tracker.

### 3.2. Adaptive Hedge Algorithm

The standard hedge algorithm [16] for decision theoretic online learning problem generates a weight distribution $w_{t}^{i}$ over all experts i∈{1,2,…,K} at frame t, where K is the number of experts. Each expert i incurs a loss $l_{t}^{i}$, and the expected loss is calculated as:

(1) $$l_{t}^{A}=\sum_{i=1}^{K}w_{t}^{i}l_{t}^{i}$$

The standard hedge algorithm introduces a new notion of regret to generate a new weight distribution over all experts for next frame t+1. The instantaneous regret to expert i is defined as:

(2) $$r_{t}^{i}=l_{t}^{A}-l_{t}^{i}$$

Its cumulative regret to expert i for frame t is:

(3) $$R_{t}^{i}=\sum_{\tau =1}^{t}r_{\tau }^{i}=R_{t-1}^{i}+r_{t}^{i}$$

The purpose of the hedge algorithm is to minimize the cumulative regret $R_{t}^{i}$ over all experts throughout the whole video frames.

Since the cumulative regret $R_{t}^{i}$ is computed by simply summing the historical regret $R_{t-1}^{i}$ and instantaneous regret $r_{t}^{i}$ as shown in Equation (3), where $R_{t-1}^{i}$ and $r_{t}^{i}$ contribute equally in the loss function, the standard hedge algorithm [16] performs not well in real-world tracking tasks as it ignores two key factors. First, the target appearance is possible to change with irregular velocity throughout a video sequence, which means that the historical regret $R_{t-1}^{i}$ should be considered with a varying proportion over time to better reflect the target state for visual tracking. Second, since each expert tracker captures a different part of the target in this work, it is less effective to utilize a fixed proportion for the historical regret over all expert trackers.

Similar to [41,44], to overcome the above two shortcomings, we propose an adaptive hedge algorithm, which is the use of an adaptive regret mechanism to determine the proportion of the historical as well as instantaneous regrets over time. Since the appearance variation of target occurs slowly in a short time period, we formulate the loss of each expert $l^{i}$ during time period ∆t via a Gaussian distribution with standard variance $\sigma_{t}^{i}$ and mean $\mu_{t}^{i}$:

(4) $$\mu_{t}^{i}=\frac{1}{∆t}\sum_{\tau =t-∆t+1}^{t}l_{\tau }^{i}$$

(5) $$\sigma_{t}^{i}=\sqrt{\frac{1}{∆t-1}\sum_{\tau =t-∆t+1}^{t}{\left(l_{\tau }^{i}-\mu_{t}^{i}\right)}^{2}}$$

The stability of expert i at frame t is decided by:

(6) $$s_{t}^{i}=\frac{\left|l_{t}^{i}-\mu_{t}^{i}\right|}{\sigma_{t}^{i}}$$

A large $s_{t}^{i}$ means that this expert varies highly and, hence, its cumulative regret should mainly depend on its historical regret. In contrast, a small $s_{t}^{i}$ means this expert tends to be more stable than the one with a larger $s_{t}^{i}$. Hence, its cumulative regret should take a large proportion on its instantaneous regret. Based on above rules, the adaptive cumulative regret for each expert is computed as follow:

(7) $$\alpha_{t}^{i}=\exp\left(-\gamma s_{t}^{i}\right)$$

(8) $$R_{t}^{i}=\left(1-\alpha_{t}^{i}\right)R_{t-1}^{i}+\alpha_{t}^{i}r_{t}^{i}$$

where γ is a parameter to control the shape of the exponential in Equation (7).

Our adaptive hedge algorithm also has the same solution form with the standard one [16]. The weight of each expert is updated for the next frame as follow:

(9) $$w_{t+1}^{i}\propto \frac{{\left[R_{t}^{i}\right]}_{+}}{c_{t}}\exp\frac{{\left(R_{t}^{i}\right)}^{2}}{2c_{t}}$$

Here ${\left[R_{t}^{i}\right]}_{+}$ denotes $max\left\{0,R_{t}^{i}\right\}$ and $c_{t}$ is a scale parameter constrained by:

(10) $$\frac{1}{K}\sum_{i=1}^{K}\exp\left(\frac{{\left({\left[R_{t}^{i}\right]}_{+}\right)}^{2}}{2c_{t}}\right)=e$$

In this work, we apply the proposed adaptive hedge algorithm to the following component trackers, respectively. In addition, different metrics used to calculate the loss of patch experts in this two component trackers are proposed.

### 3.3. Correlation Filter-Based Structural Patch Tracking (CFSP)

In CFSP, the target is split into multiple overlapped image patches $p^{i}$, i∈{1,2,…,K}, where K is the number of patches. The tracking task is then to locate these patches. During tracking, an image block $z^{i}$ with the same size of appearance template $x^{i}$ is extracted out at the location of patch $p^{i}$ in the previous frame. After that, a kernelized correlation filter (KCF) [6], which can be considered as an expert, is applied on each patch to track its position. The response map of the ith patch is calculated as:

(11) $${ℜ}_{cf}^{i}\left(z^{i}\right)=F^{-1}\left(F\left(k^{x^{i}z^{i}}\right)⊙F\left(\alpha^{i}\right)\right)$$

where the subscript cf represents the correlation filter operator. The patch $p^{i}$ in current frame is localized according to the location where the peak of the response map ${ℜ}_{cf}^{i}$. The tracking details of KCF can be found in [6].

Based on the adaptive hedge algorithm proposed in the previous section, it is natural to fuse response maps of all patches at the frame t by:

(12) $${ℜ}_{cf,t}=\sum_{i=1}^{K}w_{cf,t}^{i}{ℜ}_{cf,t}^{i}$$

where $w_{cf,t}^{i}$ is the weight of patch $p^{i}$ at frame t and $\sum_{i=1}^{K}w_{cf,t}^{i}=1$. Then at frame t, the target is located by searching the peak of the fused response map ${ℜ}_{cf,t}$.

The loss of each expert tracker need to be computed and is used by the adaptive hedge algorithm described in the above section to update the weights of all expert trackers. In CFSP, we consider two aspects for calculating the loss of each expert tracker. First, we use intensity and the smooth constraint of each patch’s response map to reflect the similarity of patch between current frame and previous frames. The peak-to-sidelobe ratio (PSR) [5] that quantifies the sharpness of the response map peak is used to estimate the intensity of response map. It is defined as:

(13) $$PSR_{t}^{i}=\frac{\max\left({ℜ}_{cf,t}^{i}\right)-mean\left({ℜ}_{cf,t}^{i}\right)}{\mathrm{var}\left({ℜ}_{cf,t}^{i}\right)}$$

where $mean\left({ℜ}_{cf,t}^{i}\right)$ and $\mathrm{var}\left({ℜ}_{cf,t}^{i}\right)$ are the mean and the standard variance of the ith patch’s response map at frame t respectively. The smooth constraint of response map (SCRM) [33] is defined as:

(14) $$SCRM_{t}^{i}={\left\|{ℜ}_{cf,t}^{i}-{ℜ}_{cf,t-1}^{i}⊕∆\right\|}_{2}^{2}$$

where ⊕ means a shift operation of the response map and ∆ denotes the corresponding shift of maximum value in response maps from frame t−1 to t. Then the normalized similarity of patch $p^{i}$ can be represented as:

(15) Sti=(PSRtiSCRMti)∑i=1K(PSRtiSCRMti)

**Figure 4:**
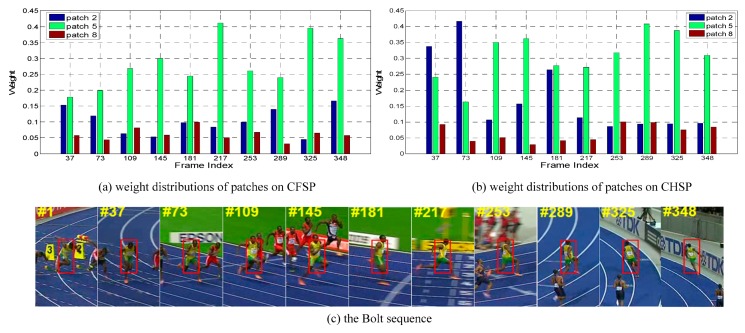
The weight distribution of component trackers in the Bolt sequence. For the sake of clarity, we only show the weight distribution of patches 2, 5, and 8. (**a**) and (**b**) are the weight distributions of the component trackers CFSP and CHSP at some frames, respectively. (**c**) shows the tracking target with red bounding box in Bolt sequence, in which the target suffers from severe deformation.

Second, we consider the displacement difference between each patch and the predicted target at frame t:

(16) $$D_{cf,t}^{i}=\frac{{\left\|dis_{cf,t}^{i}-dis_{cf,t}^{tar}\right\|}_{2}^{2}}{\sum_{i=1}^{K}{\left\|dis_{cf,t}^{i}-dis_{cf,t}^{tar}\right\|}_{2}^{2}}$$

where $dis_{cf,t}^{i}$ and $dis_{cf,t}^{tar}$ denote the displacements of corresponding patch $p^{i}$ and target with respect to frame t, respectively. The loss of the ith patch expert tracker at frame t is defined as

(17) $$l_{cf,t}^{i}=\left(1-\beta \right)\left(1-S_{t}^{i}\right)+\beta D_{cf,t}^{i}$$

where β is the trade-off between the similarity and the displacement difference. The loss calculated from Equation (17) is put into the adaptive hedge algorithm to update the weight of patch $p^{i}$ for frame t+1 in CFSP. Figure 4a illustrates the weight distribution of the sequence Bolt generated by CFSP in some frames, in which different patches have different weights. Patch 8 lies in the leg area and undergoes sever deformation. Hence, the weights of patch 8 are relatively smaller. The tracking procedure of CFSP tracker is summarized in Algorithm 1.

**Algorithm 1:** Correlation filter-based structural patch tracking**Inputs:** current weight distribution $w_{cf,t}^{1},\cdots ,w_{cf,t}^{K}$; estimated target position $pos_{t-1}$ in the previous frame; **Output:** updated weight distribution $w_{cf,t+1}^{1},\cdots ,w_{cf,t+1}^{K}$; the response map ${ℜ}_{cf,t}$ in the current frame. **Repeat:** **1:** compute correlation filter response of each patch using Equation (11); **2:** compute the fused response map ${ℜ}_{cf,t}$ using Equation (12); **3:** compute the similarity and displacement difference of each patch using Equations (13–16); **4:** compute loss of each patch tracker using Equation (17); **5:** update stability models using Equations (4) and (5); **6:** measure each patch tracker’s stability using Equation (6); **7:** update regret of each patch using Equations (1), (2), (7), and (8); **8:** update weight distribution $w_{ch,t+1}^{1},\cdots ,w_{ch,t+1}^{K}$ for each patch tracker using Equation (9);

### 3.4. Color Histogram-Based Structural Patch Tracking (CHSP)

For the overlapped image patches $p^{i}$, i∈{1,2,…,K}, we apply the same color histogram tracking method as SLC [15] to track each of them. And each color patch tracker can be regarded as an expert. Let $R_{o}^{i}$, $R_{f}^{i}$ and $R_{s}^{i}$ represent the target region, foreground and surrounding background regions of patch $p^{i}$, respectively, where the foreground region $R_{f}^{i}$ is slightly smaller than the target region $R_{o}^{i}$. Additionally, we denote $y_{u}^{i}$ as the observation of pixel u within patch $p^{i}$, which is represented by the bin of u in the color histograms. The likelihood of pixel u belongs to the region $R_{o}^{i}$ can be derived by applying Bayes rule like [27]:

(18) $$P\left(u\in R_{o}^{i}|R_{f}^{i},R_{s}^{i},y_{u}^{i}\right)\approx \frac{P\left(y_{u}^{i}|u\in R_{f}^{i}\right)P\left(u\in R_{f}^{i}\right)}{\sum_{\psi \in \left\{R_{f}^{i},R_{s}^{i}\right\}}P\left(y_{u}^{i}|u\in \psi \right)P\left(u\in \psi \right)}$$

The likelihood terms can be derived from color histogram:

(19) $$P\left(y_{u}^{i}|u\in R_{f}^{i}\right)\approx \frac{H_{f}^{i}\left(y_{u}^{i}\right)}{\left|R_{f}^{i}\right|}\;\mathrm{and}\;P\left(y_{u}^{i}|u\in R_{s}^{i}\right)\approx \frac{H_{s}^{i}\left(y_{u}^{i}\right)}{\left|R_{s}^{i}\right|}$$

where $\left|R_{f}^{i}\right|$ and $\left|R_{s}^{i}\right|$ denote the number of pixels in the foreground and surrounding background regions of patch $p^{i}$ respectively. $H_{f}^{i}\left(y_{u}^{i}\right)$ and $H_{s}^{i}\left(y_{u}^{i}\right)$ denote the color histogram over foreground and surrounding background regions. The prior probability can be approximated as:

(20) $$P\left(u\in R_{f}^{i}\right)\approx \frac{\left|R_{f}^{i}\right|}{\left|R_{f}^{i}\right|+\left|R_{s}^{i}\right|}\;\mathrm{and}\;P\left(u\in R_{s}^{i}\right)\approx \frac{\left|R_{s}^{i}\right|}{\left|R_{f}^{i}\right|+\left|R_{s}^{i}\right|}$$

Thus, the probability that pixel u belongs to the patch $p^{i}$ can be simplified to:

(21) $$P\left(u\in R_{o}^{i}\right)=P\left(u\in R_{o}^{i}|R_{f}^{i},R_{s}^{i},y_{u}^{i}\right)\approx \frac{H_{f}^{i}\left(y_{u}^{i}\right)}{H_{f}^{i}\left(y_{u}^{i}\right)+H_{s}^{i}\left(y_{u}^{i}\right)}$$

In the tracking stage, for patch $p^{i}$, we extract a rectangular searching region centered at its location in previous frame. And the response map of patch $p^{i}$ can be evaluated by using its color histogram model. Using a dense sliding-window searching way over probability map $P\left(u\in R_{o}^{i}\right)$ derived from Equation (21), we can obtain the response map of patch $p^{i}$ as follow:

(22) $${ℜ}_{ch}^{i}\left(h_{j}\right)=\frac{\sum_{u\in h_{j}}P\left(u\in R_{o}^{i}\right)}{\left|h_{j}\right|}$$

Here $\left|h_{j}\right|$ represents the number of pixels in the jth sliding window $h_{j}$, the size of which is the same as patch $p^{i}$. The location of the ith patch at this frame is estimated by searching for the peak of the response map ${ℜ}_{ch}^{i}$.

Similar as the above proposed CFSP, we also treat each patch tracker as an expert and apply the weights calculated from the adaptive hedge algorithm to fuse response maps of all patches at the frame t:

(23) $${ℜ}_{ch,t}=\sum_{i=1}^{K}w_{ch,t}^{i}{ℜ}_{ch,t}^{i}$$

where $w_{ch,t}^{i}$ is the weight of patch $p^{i}$ at frame t and $\sum_{i=1}^{K}w_{ch,t}^{i}=1$. The subscript ch denotes the color histogram operator. Then the target is located by searching the peak of the fused response map ${ℜ}_{ch,t}$.

Different from SLC [15] only exploits appearance discrimination to determine the weight of each patch, we employ both the discrimination value and displacement difference to calculate the loss of each expert tracker and put this loss into adaptive hedge algorithm to update weight. Figure 2c illustrates that our weighted method has better performance. The discrimination values [15] of patches are calculated by considering their variance ratios (VR) [45] and histogram similarities between the foreground and surrounding background regions.

The variance ratio (VR) [35,45] is to measure the discriminative power of each patch against its surrounding background. The log likelihood of pixel u within patch $p^{i}$ at frame t can be computed by using color histogram as follow:

(24) $$L_{t}^{i}\left(u\right)=\log\frac{\max\left\{H_{f,t}^{i}\left(u\right),\delta \right\}}{\max\left\{H_{s,t}^{i}\left(u\right),\delta \right\}}$$

where δ is a small value to prevent dividing by zero. The log likelihood $L_{t}^{i}$ maps the histogram into positive for colors associated with the foreground of the ith patch, and negative for colors associated with the surrounding background of the ith patch. Then the variance ratio (VR) of patch $p^{i}$ at frame t can be computed as:

(25) $$VR_{t}^{i}\left(L_{t}^{i},H_{f,t}^{i},H_{s,t}^{i}\right)=\frac{\mathrm{var}\left(L_{t}^{i};\left(H_{f,t}^{i}+H_{s,t}^{i}\right)/2\right)}{\mathrm{var}\left(L_{t}^{i};H_{f,t}^{i}\right)+\mathrm{var}\left(L_{t}^{i};H_{s,t}^{i}\right)}$$

where var(L;H) defines the variance of L(u) with respect to the color histogram H(u) and is calculated as:

(26) $$\mathrm{var}\left(L;H\right)=\sum_{u}H\left(u\right)L^{2}\left(u\right)-{\left[\sum_{u}H\left(u\right)L\left(u\right)\right]}^{2}$$

In Equation (25), the denominator is small when the log likelihood values of pixels in the patch and background classes are tightly clustered, while the numerator is large when the two clusters are widely separated. Thus, patches with large variance ratio show stronger discriminative power to separate the foreground and surrounding background.

Moreover, less similarity of histograms between foreground and surrounding background can readily distinguish the target from its surroundings. Therefore, the Bhattacharyya distance can be exploited:

(27) $$\rho_{t}^{i}\left(H_{f,t}^{i},H_{s,t}^{i}\right)=\sum_{u}\sqrt{H_{f,t}^{i}\left(u\right)H_{s,t}^{i}\left(u\right)}$$

Thus, the normalized discrimination of patch $p^{i}$ can be defined as:

(28) dti=VRtiρti∑i=1K(VRtiρti)

Therefore, the loss of the ith patch expert at frame t is defined as:

(29) $$l_{ch,t}^{i}=\left(1-\beta \right)\left(1-d_{t}^{i}\right)+\beta D_{ch,t}^{i}$$

where $D_{ch,t}^{i}$ denotes the displacement difference between the ith patch and the predicted target in CHSP at frame t:

(30) $$D_{ch,t}^{i}=\frac{{\left\|dis_{ch,t}^{i}-dis_{ch,t}^{tar}\right\|}_{2}^{2}}{\sum_{i=1}^{K}{\left\|dis_{ch,t}^{i}-dis_{ch,t}^{tar}\right\|}_{2}^{2}}$$

Figure 4b displays the weight distribution of the sequence Bolt generated by CHSP at some frames. Similar as CFSP, different patches also have different weights and patches 5 and 8 have obvious distinction, of which the patch 5 is the middle part of the body whereas the patch 8 is the leg area. The leg area contains more background interference and has poor motion consistency with the body part. The tracking procedure of CHSP tracker is summarized in Algorithm 2.

**Algorithm 2:** Color histogram-based structural patch tracking**Inputs:** current weight distribution $w_{ch,t}^{1},\cdots ,w_{ch,t}^{K}$; estimated target position $pos_{t-1}$ in the previous frame; **Output:** updated weight distribution $w_{ch,t+1}^{1},\cdots ,w_{ch,t+1}^{K}$; the response map ${ℜ}_{ch,t}$ in the current frame. **Repeat:** **1:** compute color histogram response of each patch using Equation (22); **2:** compute the response map ${ℜ}_{ch,t}$ using Equation (23); **3:** compute the discrimination and displacement difference of each patch using Equations (24)–(28) and (30); **4:** compute loss of each patch tracker using Equation (29); **5:** update stability models using Equations (4) and (5); **6:** measure each patch tracker’s stability using Equation (6); **7:** update regret of each patch using Equations (1), (2), (7) and (8); **8:** update weight distribution $w_{cf,t+1}^{1},\cdots ,w_{cf,t+1}^{K}$ for each patch tracker using Equations (9);

### 3.5. Response Maps Fusion between CFSP and CHSP

To complement the strengths of CFSP and CHSP, inspired by [15], we combine their response maps in a selective strategy as well. Different from LGCmF [15] using the peak value of response map in the global layer tracker to analyzing the confidence, we apply the online support vector machine (SVM) classifier on both the tracking results of CHSP and CFSP to evaluate their confidences. Specifically, we first use the SVM classifier on the tracking results of CFSP and CHSP to obtain the confidence scores $C_{cfsp}$ and $C_{chsp}$. When $C_{cfsp}$ or $C_{chsp}$ are larger than the predefined thresholds $T_{cfsp}$ or *T_chsp_*, we consider that the CFSP or the CHSP tends to be credible. Therefore, the merging factor ($\eta_{cfsp}$ or $\eta_{chsp}$) can be picked according to the credibility of the two component trackers:

(31) $$ℜ=\eta {ℜ}_{ch}+\left(1-\eta \right){ℜ}_{cf}$$

where ${ℜ}_{ch}$ and ${ℜ}_{cf}$ are the response maps of CHSP and CFSP, respectively. $\eta =\eta_{cfsp}\;\mathrm{or}\;\eta_{chsp}$ is the merging factor that is chosen based on the confidences of CFSP and CHSP. If the confidence scores $C_{cfsp}$ and $C_{chsp}$ are both below the thresholds $T_{cfsp}$ and $T_{chsp}$, we consider that the CFSP and CHSP are unreliable at this frame. Similar as [15,17], a re-detection process using the SVM classifier is performed by drawing dense candidates around the searching region. In this case the detected result of the SVM can be adopted only if its maximum detecting score $\max\left(C_{svm}\right)$ is above a threshold $T_{svm}$ to guarantee the accuracy. Once $\max\left(C_{svm}\right)<T_{svm}$, the re-detected result is given up and we select the $\eta_{cfsp}$ as the merging factor in Equation (31). At this time the target usually suffers from partial occlusion or severe deformation, we trust the CFSP tracker more as its performance is more robust and accurate compared to the CHSP tracker, which is illustrated in experiment section. The tracking procedure of final CSPRF tracker is summarized in Algorithm 3.

**Algorithm 3:** Complementary structural patches response fusion tracking (CSPRF)**Inputs:** the responses of the CFSP and CHSP ${ℜ}_{cf,t}$, ${ℜ}_{ch,t}$; estimated target position $pos_{t-1}$ in the previous frame; **Output:** estimated current target position $pos_{t}$. **Repeat:** **1:** obtain the confidence scores $C_{cfsp}$ and $C_{chsp}$ using the SVM classifier on the tracking results of CFSP and CHSP. **2:**
**if**
*C_chsp_* ≥ *T_chsp_*
**then** **3:** set $\eta =\eta_{chsp}$ and compute the current target position $pos_{t}$ using Equation (31); **4: else if**
$C_{cfsp}\geq T_{cfsp}$
**then** **5:** set $\eta =\eta_{cfsp}$ and compute the current target position $pos_{t}$ using Equation (31); **6:** **else** **7:**  use the online SVM classifier to draw dense candidates around $pos_{t-1}$ and obtain the detecting scores $C_{svm}$ of all candidate samples; **8:**  **if**
$\max\left(C_{svm}\right)\geq T_{svm}$
**then** **9:**   current target position $pos_{t}=\arg\max(C_{svm})$; **10:**  **else** **11:**   set $\eta =\eta_{cfsp}$ and compute the current target position $pos_{t}$ using Equation (31); **12:**  **end** **13:** **end** **14: end**

### 3.6. Update Scheme

To adapt to the target appearance variations, we need to update CFSP tracker, CHSP tracker and the SVM classifier. For CFSP tracker, we incrementally update the correlation filter of each patch when its response map peak $\max\left({ℜ}_{cf,t}^{i}\right)$ at frame t is above the threshold $T_{peak}$:

(32a) $${\tilde{\alpha }}_{t}^{i}=\{\begin{array}{cc} \left(1-\xi \right){\tilde{\alpha }}_{t-1}^{i}+\xi \alpha_{t}^{i}, & if\;\max\left({ℜ}_{cf,t}^{i}\right)\geq T_{peak}\\ {\tilde{\alpha }}_{t-1}^{i}, & otherwise \end{array}$$

(32b) $${\tilde{x}}_{t}^{i}=\{\begin{array}{cc} \left(1-\xi \right){\tilde{x}}_{t-1}^{i}+\xi x_{t}^{i}, & if\;\max\left({ℜ}_{cf,t}^{i}\right)\geq T_{peak}\\ {\tilde{x}}_{t-1}^{i}, & otherwise \end{array}$$

Here ξ is the learning rate. For CHSP tracker, the color histograms of each patch are update as follow:

(33) $${\tilde{H}}_{c,t}^{i}=\{\begin{array}{cc} \left(1-\tau \right){\tilde{H}}_{c,t-1}^{i}+\tau H_{c,t}^{i}, & if\;d_{t}^{i}\geq T_{dis}\\ {\tilde{H}}_{c,t-1}^{i}, & otherwise \end{array}$$

where τ is the learning rate and $H_{c,t}^{i}\in \left\{H_{f,t}^{i},H_{s,t}^{i}\right\}$ indicates the learned color histograms of foreground and surrounding background regions of patch $p^{i}$ at frame t. $d_{t}^{i}$ is the discrimination value of patch $p^{i}$ at frame t computed from Equation (28), and $T_{dis}$ is the predefined threshold.

For the SVM classifier, it is updated only when $C_{cfsp}\geq T_{cfsp}$ or $C_{chsp}\geq T_{chsp}$, since at this time we consider the current tracking result is credible. We incrementally update the SVM classifier by applying the passive-aggressive algorithm [46] efficiently, which is similar to [17].

### 3.7. Scale Estimation

Similar to the DSST tracker [21], we first localize the target in a new frame and subsequently estimate scale variation. We train a one-dimensional correlation filter to perform scale estimation. A scaling set $S=\left\{a^{n}|n\in \left\{\left\lfloor -\frac{N_{s}-1}{2}\right\rfloor ,\ldots ,\left\lfloor \frac{N_{s}-1}{2}\right\rfloor \right\}\right\}$ is built, where a and $N_{s}$ denote the scale parameter and the number of scales respectively. Let M×N be the target size in the current frame and for each scale s∈S, an image patch $z_{s}$ of size sM×sN centered at the target location is extracted to construct a feature pyramid. We exploit the correlation filter on these image patches $z_{s}$ with corresponding to one dimensional Gaussian regression label $y_{s}$. The estimated scale is derived as:

(34) $$s_{opt}=\arg\max\left\{f\left(z_{s}\right)|s\in S\right\}$$

where $s_{opt}$ is the maximum value of the scale correlation response. This implementation details can refer to [21].

## 4. Experimental Results

We first evaluate our complementary structural patches response fusion tracker (CSPRF) by comparing with others state-of-the-art trackers on OTB2013 and OTB2015. Then, the performance comparison of the LGCmF with our CSPRF is conducted. After that, to validate the effectiveness of two component trackers (CFSP and CHSP), we compare them with several relevant tracking algorithms, respectively. Finally, we conduct comparative experiments on VOT2016 [12].

### 4.1. Experimental Setup

We conducted our experiments on OTB2013 [10] and OTB2015 [11] benchmarks. All these sequences cover 11 challenging attributes: background clutters (BC), deformation (DEF), fast motion (FM), scale variation (SV), out-of-plane rotation (OPR), motion blur (MB), out-of-view (OV), in-plane rotation (IPR), illumination variation (IV), occlusion (OCC), and low resolution (LR). The tracking methods are evaluated by the following metrics: center location error (CLE), distance precision rate (DP), and overlap success rate (OS). The CLE is defined as the average Euclidean distance between the ground truth and the estimated center location of the target. The DP is computed as the percentage of frames where CLE is smaller than a specified threshold. The OS indicates the percentage of frames whose overlap ratio between the estimated bounding box and the ground truth bounding box surpasses a certain threshold. Following the evaluation protocol [10,11], we set the two preset thresholds of the DP and OS to 20 pixels and 0.5 in overall experiments, respectively. In addition, experimental results are reported using the precision plots and success plots under one-pass evaluation (OPE) as in [10,11]. In success plots, the area under the curve (AUC) is adopted to rank the compared trackers in the legend.

Besides OTB2013 and OTB2015, we also implement comparative experiments on VOT2016 [12]. This dataset consists of 60 challenging sequences. The performance is evaluated both in terms of robustness, accuracy and expected average overlap (EAO). The robustness calculates the average number of tracking failures over all sequences. The accuracy computes the average overlapping ratio between the estimated bounding box and the ground truth. EAO ranks the overall performance which takes both accuracy and robustness into account. Readers can refer to [12] for details.

Our methods are implemented in MATLAB 2014a (MathWorks, Natick, MA, USA) for learning and tracking process and C++ for feature extraction. The source codes of compared tracking algorithms are offered by authors, whose parameters are at default values. All the experiments are run on a PC with an AMD A10-5800K 3.8GHz CPU and 8 GB of RAM (Advanced Micro Devices, Sunnyvale, CA, USA).

### 4.2. Implementation Details

Let $M_{o}\times N_{o}$ represent the size of the target bounding box. The global target is divided into 3×3 overlapped patches by taking the patch size and step length as $\left(\frac{M_{o}}{2},\frac{N_{o}}{2}\right)$, that is to say, the parameter K=9. The time period ∆t in Equations (4) and (5) is set to five frames and the scale factor γ in Equation (7) is set to 10. The *β* in Equations (17) and (29) is set to 0.5. For the component tracker CFSP, the histogram of the oriented gradient (HOG) [13] and color names (CN) [20] are applied as the feature representation. The searching window size of M×N is set to four times the patch size. The learning rate ξ in Equation (32) is set to 0.01 and the threshold $T_{peak}=0.16$.

For the component tracker CHSP, the surrounding background region $R_{s}$ is an expanded region of patch with $\frac{1}{2}\left(\frac{M_{o}}{2}+\frac{N_{o}}{2}\right)$ as the length and width, while the foreground region $R_{f}$ is set to 0.8 times the patch size $R_{o}$. In Equation (24), $\delta =10^{-3}$. The learning rate τ in Equation (33) is set to 0.04 and the threshold $T_{dis}$ is set to 0.5/2.5 for gray/color image sequences. For CSPRF, the thresholds $T_{cfsp}$, *T_chsp_* and $T_{svm}$ are set to 0, 0, and 0.5, respectively. The merging factors $\eta_{cfsp}$ and $\eta_{chsp}$ are set to 0.6 and 0.3. The SVM classifier is trained by densely drawing samples from a searching window centered at the global target location. The samples with positive label are selected when their overlap ratios with the global target bounding box are above 0.6, and for the samples with negative label, their overlap ratios are below 0.2. For scale estimation, the parameters are the same as the DSST [21] tracker. We keep the above parameters fixed throughout all of the experiments and our proposed CSPRF tracker runs at an average of 5.1 frames per second (FPS).

### 4.3. Performance Evaluation of the CSPRF Tracker on OTB2013 and OTB2015

Our proposed CSPRF tracker is compared with 10 state-of-the-art trackers including KCF [6], MEEM [4], DSST [21], Staple [14], Staple_CA [8], CSR-DCF [28], SRDCF [7], SAMF [47], LCT+ [17] and RPT [34]. In above trackers, KCF, DSST, and SRDCF are the correlation filters-based trackers. Staple, Staple_CA, CSR-DCF and SAMF introduce color feature as an effective complement to the HOG feature. RPT is the part-based tracker and MEEM is the tracker that uses multiple online SVM classifiers.

#### 4.3.1. Quantitative Evaluation

**Figure 5:**
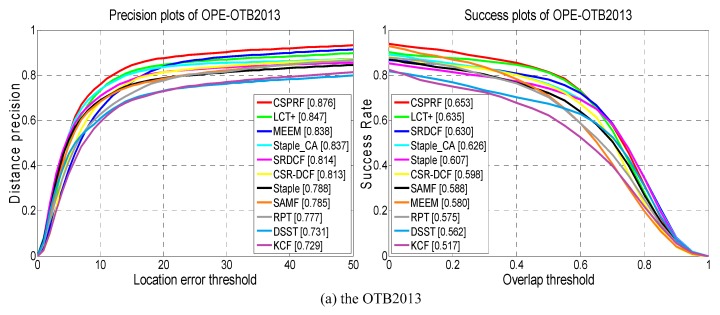

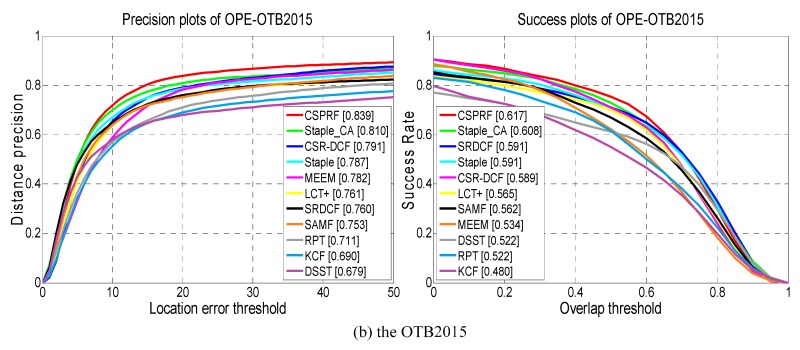
Quantitative evaluation over the OTB2013 and OTB2015 datasets. Precision and success plots using the one-pass evaluation (OPE). The legend of precision plots shows the average distance precision rates (DP) at 20 pixels, and the legend of success plots contains the overlap success scores (OS) with the area under the curve (AUC).

Figure 5 and Table 1 show overall comparisons between our CSPRF tracker and other 10 trackers on OTB2013 and OTB2015 datasets. It is easily to observe that our CSPRF tracker performs favorably against the compared trackers on both datasets. For the OTB2013 dataset as shown in Figure 5a, the proposed CSPRF tracker achieves the best overall performance both in precision and success plots with a DP score of 87.6% and an AUC score of 65.3%, outperforming the second best tracker LCT+ by 2.9% and 1.8%. For OTB2015 dataset as illustrated in Figure 5b, the CSPRF performs best with a DP score of 83.9% on the precision plot and an AUC score of 61.7% on the success plot, and outperforms the second best Staple_CA by 2.9% and 0.9%, respectively. In contrast to the Staple_CA that only promotes the correlation filter module of Staple, our method improves both the correlation filter and color histogram modules of Staple and, hence, obtains better performance than Staple_CA. Additionally, compared with the Staple tracker, our approach achieves gains of 8.8% and 5.2% in the DP score and 4.6% and 2.6% in the AUC score on both OTB2013 and OTB2015, respectively.

Table 1 reports the mean OS (%), median DP (%), median OS (%) and median CLE (pixels) over the OTB2013 and OTB2015 datasets. Our tracker obtains the best results in above three evaluation metrics except that its median CLEs with 6.39 on OTB2013 and 7.10 on OTB2015 are slightly lower than the SRDCF and Staple_CA by 1.57 and 0.01, respectively.

#### 4.3.2. Attribute-Based Evaluation

**Figure 6:**
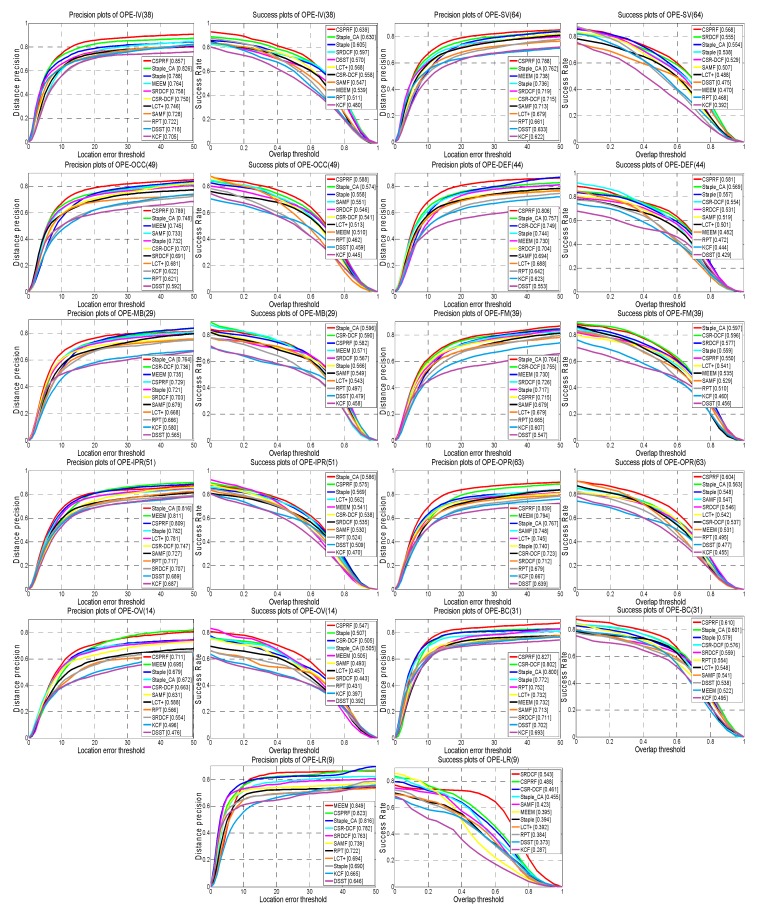
The precision plots and the success plots with 11 attributes on OTB2015. The legend of precision plot contains the average DP score at 20 pixels while the legend of success plot contains the area under the curve (AUC) score for each tracker. The number of sequences for each attribute is shown in brackets.

To facilitate analyzing the strength and weakness of our method in various aspects, we further evaluate the trackers on datasets with 11 attributes. Figure 6 shows the precision and success plots of all compared trackers on OTB2015 with various attributes. Among them, our tracker ranks the best within seven out of 11 attributes including OPE, SV, OCC, DEF, OPR, OV and BC, and achieves a top three performance in terms of IPR and LR. This is attributed to our proposed complete structural patch tracking strategy and the novel updated weight strategy, which can fully emphasize valid cues of the target. Especially, our tracker makes a large margin in terms of BC, DEF, and OCC in the precision plots. This illustrates that our tracker has the distinct advantage in dealing with the background clutter, deformation, and occlusion.

Table 2 reports the mean DP scores of compared trackers over all 11 attributes on the OTB2013. Our CSPRF tracker obtains the best performance within seven out of 11 attributes including IV, SV, OCC, DEF, OPR, OV, and BC, and achieves the second best performance in IPR with the mean DP score of 82.8%. From Figure 6 and Table 2, it demonstrates that our proposed CSPRF obtains competitive tracking performance against the other state-of-the-art trackers in these challenging attributes.

#### 4.3.3. Qualitative Evaluation

**Figure 7:**
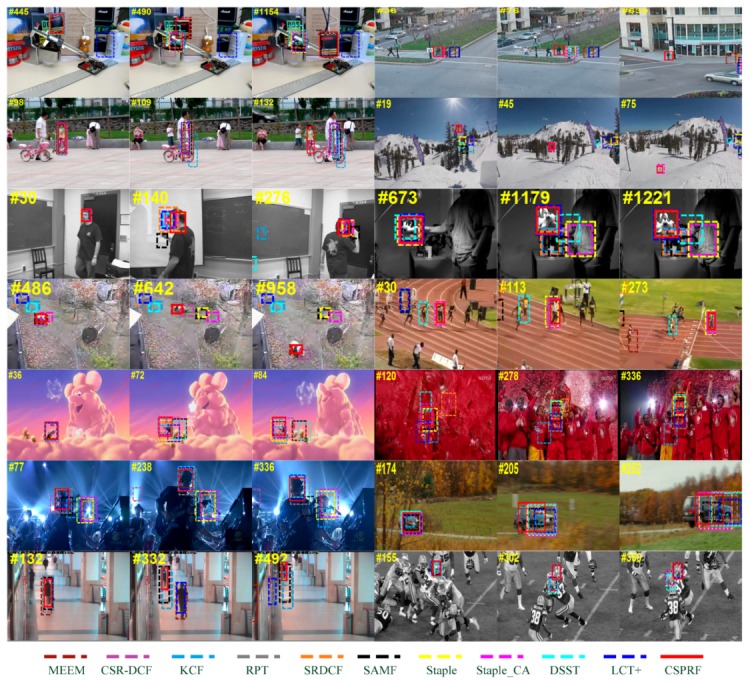
Qualitative evaluation of the proposed algorithm with 10 state-of-the-art methods on 14 challenging video sequences (from left to right and from top to bottom are Box, Human3, Girl2, Skiing, Freeman1, Sylvester, Panda, Bolt2, Bird2, Soccer, Shaking, CarScale, Walking2, and Football, respectively).

Figure 7 illustrates the qualitative comparison of our CSPRF tracker with mentioned 10 trackers on 14 challenging sequences. From these figures, it is clearly observed that our method performs well in all these challenging sequences.

**Occlusion.** In the Box sequence, the LCT+ quickly drifts to the similar background area from the beginning, and the target is gradually occluded by the Vernier caliper from the 445th frame. When the target reappears in the 490th frame, only our CSPRF, SAMF and MEEM successfully track it while other trackers still stay on the obstruction (Vernier caliper). In the Human3 sequence, LCT+, SAMF, KCF, MEEM, and RPT fail to track the target in the 36th frame. After a short partially occluded duration, all other trackers lose the target as well, only our tracker sticks on it throughout the sequence. In addition, in the Girl2 sequence, only our method can effectively capture it again when the target reappears, while all other compared trackers drift toward the distracter that has the similar appearance as the girl. Here, the success of our tracker is mainly attributed to the confidence updating strategy and the online re-detection mechanism.

**Rotation.** The target undergoing the in-plane or out-of-plane rotation often causes the variation of target appearance, which will increase the tracking difficulty. In the Skiing sequence, since the target keeps rotating in consecutive frames, most of trackers lose the target in the 19th frame. Only our CSPRF, Staple_CA, and MEEM successfully track the target in the entire tracking period. In the Freeman1 sequence, all the trackers perform well at the beginning, such as frame 30. The target undergoes the out-of-plane rotation at the 140th frame, our tracker and Staple get right estimates in location and scale, and the other trackers all drift to the face of the man, SAMF even loses the target completely. At frame 276, KCF and DSST also lose the target. Another example where the rotation is the main challenge is Sylvester sequence. At frame 1179, only MEEM, LCT+, RPT, and our CSPRF locate the target while other trackers fail to track the target.

**Deformation.** In Panda sequence, the target suffers from severe deformation. LCT+, SRDCF, KCF, DSST and RPT lose the target at frame 486 and more trackers drift to the background when the panda passes by the tree, whereas our CSPRF, MEEM still track the target (e.g., frames 642, 958). Although the Staple_CA can track the target, it gets inaccurate target location. In Bolt2 sequence, the target undergoes severe deformation as well. Others trackers fail to track the target form the beginning, only our CSPRF, CSR-DCF, Staple and Staple_CA successfully track the target in the whole tracking period. In the Bird2 sequence, many trackers obtain inaccurate target location when the bird turns around at frame 72, and SAMF and DSST fail to track the target at this time. Only our CSPRF, MEEM, and Staple_CA obtain the accurate results in the overall tracking process.

**Background clutter.** The existence of similar-appearing objects to the target in the background makes it challenging to distinguish the target from the background and accurately locate the target. In the Soccer sequence, among all 11 compared trackers, the KCF, LCT+, SRDCF, MEEM, and DSST lose the target at frame 120, and RPT, Staple, and SAMF obtain inaccurate results in terms of location and scale. Only our CSPRF, Staple_CA, and CSR-DCF get the reliable tracking results both in scale and location during the entire tracking period. In the Shaking sequence, Staple_CA, SRDCF, and KCF fail to locate the target and drift to the distracters in the 77th frame. At frame 238, CSR-DCF and SAMF lose the target as well. Only our CSPRF, MEEM, LCT+, and DSST successfully track the target. Although RPT can locate the target, it obtains an incorrect scale estimate. In the Football sequence, most of the compared trackers drift to the distracters at frame 302, only our method, LCT+, MEEM, and SRDCF stick on the target and favorably track the target over all frames.

**Scale variation.** Due to the KCF and MEEM without handling the scale variation, they do not perform well when the target undergoes large scale variation. The targets in the CarScale and Walking2 sequences undergo the scale variation from beginning to end. In the CarScale sequence, MEEM and KCF obtain inaccurate tracking results in scale in the 174th frame. At frame 205, only our tracker obtains accurate results in scale and location, while many other trackers focus on the head of the car. In the Walking2 sequence, MEEM, RPT, KCF, and SAMF do not perform well in scale at frame 132. MEEM and LCT+ eventually drift away to the distracter at frame 332. Our tracker with others trackers, including DSST, SRDCF, Staple, Staple_CA, and CSR-DCF, all perform well in scale and location in the whole tracking period.

### 4.4. Performance Comparison of LGCmF with CSPRF

Since LGCmF exploits the block tracking and response fusion strategies as well, we compare our CSPRF tracker with the LGCmF tracker on OTB2015. Table 3 shows comprehensive performance comparison between these two trackers. Our CSPRF outperforms LGCmF in all evaluation criteria. The reason that our method obtains better results lies in the fact that we adopt a complete block tracking strategy, a novel adaptive hedge algorithm to update the weights and efficient loss metrics in both component trackers.

**Figure 8:**
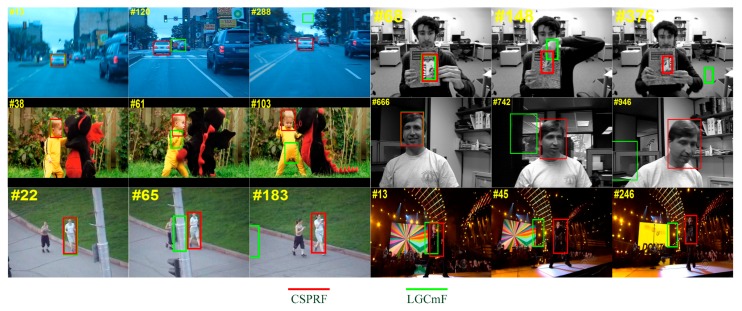
Visualization of the tracking results of LGCmF and CSPRF trackers on six challenging sequences. (from left to right and from top to bottom are BlurCar3, ClifBar, DragonBaby, Dudek, Jogging2, Singer2).

Figure 8 visualizes the tracking results of the LGCmF tracker with our CSPRF tracker on six challenging sequences. CSPRF tracker can perform well when the target objects undergo in-plane rotation (ClifBar), motion blur (BlurCar3), out-of-plane rotation (DragonBaby), background clutter (Dudek), occlusion (Jogging2), and illumination variation (Singer2), whereas the LGCmF fails in all of these sequences.

### 4.5. Performance Evaluation of Component Trackers CFSP, CHSP

To better understand the improvements of the two component trackers of our CSPRF, in this section we carry out experimental evaluations by comparing with some relevant trackers on OTB2013 and OTB2015.

We compare the tracking performance of CFSP with four relevant trackers, including KCF [6], Staple_cf_ [14], RPT [34], and SAMF [47]. Among them, Staple_cf_ is the part of Staple based on the correlation filter. KCF is the baseline tracker which is used to track each patch in our CFSP. SAMF also employs color names as complementary feature which is the same as our CFSP. In addition, RPT attempts to find the motion trajectory and trackability of random parts.

**Figure 9:**
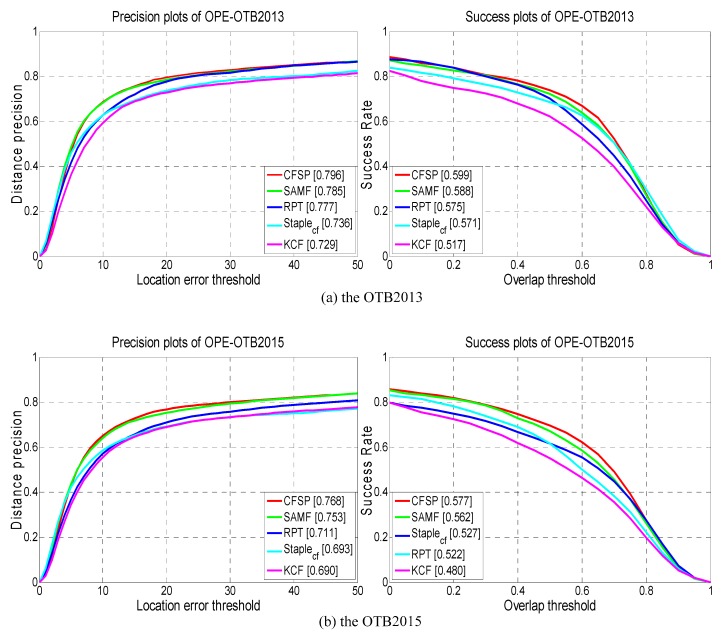
Comparison of the CFSP with four relative trackers on OTB2013 and OTB3015. The legend of the precision plot contains the average DP score at 20 pixels while the legend of success plot contains the area under the curve (AUC) score for each tracker.

Figure 9 shows the precision and success plots on the OTB2013 and OTB2015. Overall, our CFSP tracker performs favorably and achieves the best results against the other compared trackers. This demonstrates the effectiveness of the adaptive hedge algorithm and loss terms in CFSP. Specifically, our CFSP significantly improves the Staple_cf_ with gains of 6.0% in the DP score and 2.8% in the AUC score on OTB2013, and with gains of 7.5% in the DP score and 5.0% in the AUC score on OTB2015. Additionally, RPT is also a part of the tracking algorithm based on correlation filters, and our CFSP outperforms the RPT with gains of 1.9% and 5.7% in the DP scores and 2.4% and 5.5% in the AUC scores on OTB2013 and OTB2015, respectively.

We evaluate our component tracker CHSP on OTB2013 and OTB2015 with four relevant trackers including DAT [27], Staple_ch_ [14], PPT [48], and SLC [15]. The Staple_ch_ only contains the part of Staple based on the color histogram. Both PPT and SLC employ part-based color histogram appearance models, while DAT exploits the holistic color histogram appearance model.

**Figure 10:**
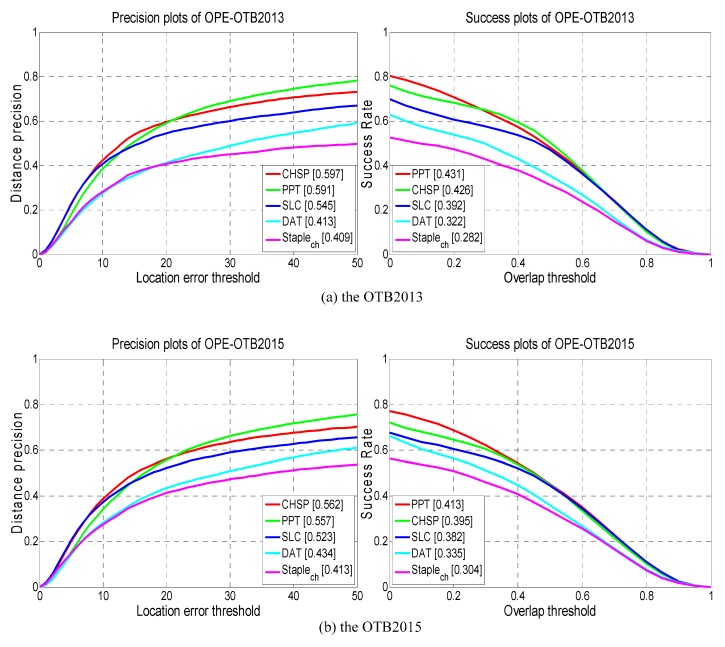
Performance comparison of the CHSP with several relative trackers on OTB2013 and OTB3015. The legend of precision plot contains the average DP score at 20 pixels while the legend of success plot contains the area under the curve (AUC) score for each tracker.

Figure 10 visualizes the precision and success plots of our CHSP with four compared trackers. From the figures, we can discover that our CHSP achieves competitive performance against the relevant trackers. Our CHSP is mere inferior to the PPT with losses of 0.5% and 1.8% in the AUC on OTB2013 and OTB2015, respectively. The tracking performance of Staple_ch_ is not satisfactory in the overall evaluation, which ranks at the bottom. Although DAT using the holistic color histogram models owns the similar tracking idea with Staple_ch_, DAT performs better because of adding analysis of the distracters in the tracking process. Specifically, our CHSP outperforms the DAT and Staple_ch_ with gains of 18.4% and 18.8% in the DP scores and 10.4% and 14.4% in the AUC scores on OTB2013 respectively, and with gains of 12.8% and 14.9% in the DP scores and 6.0% and 9.1% in the AUC scores on OTB2015, respectively. SLC employ the same block framework as our CHSP, and its tracking performance has been significantly improved compared to Staple_ch_ and DAT. Our CHSP tracker outperforms the SLC with gains of 5.2% in the DP score and 3.4% in the AUC score on OTB2013, and outperforms the SLC with gains of 3.9% in the DP score and 1.3% in the AUC score on OTB2015. This demonstrates the advantages of the adaptive hedge algorithm and loss terms in CHSP.

### 4.6. Performance Evaluation of the CSPRF Tracker on VOT2016

We compare our CSPRF tracker with eight state-of-the-art trackers, including CSR-DCF [28], DAT [27], DSST [21], HCF [40], KCF [6], SRDCF [7], Staple [14], and STRCF [9]. Table 4 lists the tracking results on VOT2016. Our CSPRF performs the second best EAO score of 0.307, only below the CSR-DCF with the best score of 0.332. According to the analysis of [12], the EAO score of our CSPRF is 0.307 which outperforms the definition of the strict state-of-the-art bound 0.251 by 5.6%, and thus it can be regarded as state-of-the-art. And CSPRF achieves some improvement against Staple by a gain of 1.2% in the EAO metric. As for accuracy and robustness, our CSPRF ranks within top three on both two metrics, which demonstrate that our tracker achieves competitive performances against compared trackers.

## 5. Conclusions

Based on the success of the Staple tracker, we extend it and propose a novel structural patch complementary tracking algorithm in this paper. We firstly present an adaptive hedge algorithm to overcome the disadvantage of the fixed percentage factor used in the standard hedge algorithm. In the component trackers CHSP and CFSP, we design two reliable loss measurement methods of structural patches, respectively, by which the adaptive hedge algorithm can reliably weigh patches to combine their response maps. The final CSPRF tracker is formulated by selectively merging the response maps of component trackers CHSP and CFSP. In addition, when both of component trackers CHSP and CFSP are unreliable, an online SVM detector is activated to rediscover the target in an extended searching area. Extensive experimental results on OTB2013, OTB2015, and VOT2016 show that the proposed algorithm CSPRF performs favorably against the state-of-the-art trackers in terms of accuracy and robustness. Meanwhile, the CSPRF and the component tracker CHSP have some tracking performance improvements in comparison with the LGCmF and its local layer tracker SLC, respectively. Moreover, the superiorities of two component trackers CHSP and CFSP are justified by comparing with some relevant trackers, in which the CHSP and CFSP have greatly improved in comparison with Staple_ch_ and Staple_cf_, respectively.

## Figures and Tables

**Table 1:** Overall performance on the OTB2013 (I) and OTB2015 (II) datasets with the representative mean overlap success (OS) rate at threshold of 0.5, median overlap success (OS) rate, median distance precision (DP) rate, and median center location error (CLE). Best: bold; second best: underline.

		CSPRF	LCT+	DSST	Staple_CA	Staple	SAMF	SRDCF	RPT	KCF	CSR-DCF	MEEM
Meam OS (%)	I	**81.4**	81.2	67.3	76.1	74.2	72.2	78.1	70.2	62.1	75.6	70.8
	II	**75.4**	70.1	61.3	72.8	70.4	67.0	71.2	61.6	55.1	71.2	62.2
Median OS (%)	I	**82.5**	82.3	68.0	77.2	75.1	73.4	78.8	71.9	63.7	76.9	72.9
	II	**76.6**	71.3	62.2	74.5	71.8	68.7	72.3	63.6	56.9	72.3	64.5
Median DP (%)	I	**89.1**	86.1	75.1	85.0	80.2	80.6	82.7	80.5	75.5	83.0	86.7
	II	**85.6**	78.2	69.8	82.7	80.4	77.6	78.3	74.0	71.7	81.5	81.0
Median CLE (pixel)	I	6.39	7.23	12.2	7.27	8.42	8.72	**4.82**	8.26	11.4	7.98	7.50
	II	7.10	9.13	13.1	**7.09**	8.35	9.43	7.75	11.3	14.7	8.50	9.92

**Table 2:** Distance precision scores (%) at a threshold of 20 pixels in terms of individual attributes on the OTB2013. Best: bold, second best: underline.

	CSPRF	LCT+	DSST	Staple_CA	Staple	SAMF	SRDCF	RPT	KCF	CSR-DCF	MEEM
IV(25)	**84.5**	79.2	72.4	80.1	74.2	70.6	71.3	74.1	70.7	71.3	76.9
SV(28)	**84.4**	75.7	71.5	80.5	73.6	73.0	77.1	74.0	65.5	70.0	70.0
OCC(29)	**87.0**	84.6	70.0	80.6	78.3	84.5	81.2	73.5	73.1	79.0	81.6
DEF(19)	**90.1**	87.0	66.3	83.9	78.8	81.9	79.5	72.8	74.6	82.6	84.6
MB (12)	71.1	66.5	54.0	**78.5**	70.8	61.3	72.9	72.6	60.5	72.4	71.3
FM (17)	71.6	66.3	51.8	**76.6**	66.1	65.4	73.0	67.7	57.0	73.2	74.1
IPR (31)	82.8	80.2	75.2	**83.9**	78.8	72.2	75.0	77.7	70.8	74.6	80.9
OPR(39)	**87.6**	84.9	72.0	82.3	77.4	77.8	78.7	77.0	71.5	78.5	84.9
OV (6)	**76.6**	72.8	51.4	69.7	65.0	63.5	70.6	67.8	64.8	66.2	74.4
BC (21)	**84.0**	79.3	69.2	79.0	74.9	71.7	72.7	78.4	72.3	78.8	79.8
LR (4)	80.4	71.7	69.0	97.2	69.5	65.0	76.9	78.1	62.9	65.3	**98.7**

**Table 3:** Performance comparison of LGCmF with CSPRF on OTB2015 with the representative mean distance precision (DP) rate at the threshold of 20 pixels, mean overlap success (OS) rate at the threshold of 0.5, median distance precision (DP) rate, median overlap success (OS) rate, median center location error (CLE), and the area under the curve (AUC). Best: bold.

	Mean DP (%)	Mean OS (%)	Median DP (%)	Median OS (%)	Median CLE	AUC
LGCmF	80.6	72.2	82.4	74.1	8.35	59.8
CSPRF	**83.9**	**75.4**	**85.6**	**76.6**	**7.10**	**61.7**

**Table 4:** Performance comparison of different trackers on VOT2016 with expected average overlap (EAO), accuracy and robustness. Best: bold, second best: underline.

	CSPRF	CSR-DCF	DAT	DSST	HCF	KCF	SRDCF	Staple	STRCF
EAO	0.307	**0.332**	0.217	0.181	0.220	0.194	0.246	0.295	0.252
Accuracy	0.53	0.52	0.47	0.53	0.45	0.49	0.53	**0.54**	0.51
Robustness	0.97	**0.90**	1.72	2.52	1.42	2.03	1.5	1.35	1.35
